# Adult‐born neurons without LRP1 evade apoptosis during neurogenesis and impair hippocampal memory

**DOI:** 10.1002/alz70861_108175

**Published:** 2025-12-23

**Authors:** Naomi Sayre

**Affiliations:** ^1^ University of Texas Health San Antonio, San Antonio, TX USA

## Abstract

**Background:**

Adult hippocampal neurogenesis is essential to memory and learning, and is disrupted in Alzheimer's disease. Mechanisms to elucidate the the factors which influence adult neurogenesis remain an active area of exploration. We have discovered that low‐density lipoprotein receptor 1 (LRP1) modulates adult neural stem cell biology. Notably, LRP1 is a considerable target of interest in Alzheimer's disease due to its interactions with amyloid beta, ApoE4, and tau. We tested whether disruption of LRP1 function impairs adult neurogenesis.

**Method:**

We employed triple‐transgenic mice, to inducibly knockout floxed LRP1 specifically in Nestin‐CreERt2‐expressing adult neural stem cells of 3‐month‐old mice after treatment with tamoxifen. Tamoxifen treatment also induced expression of stop‐floxed td‐tomato to allow tracking of neurons. We measured the effect on behavior and neurogenesis at 4 months of age and 9 months of age.

**Result:**

We found that by 9 months of age, mice lacking LRP1 in adult neural stem cells showed impaired hippocampal memory as measured via the Barnes Maze and Y maze. We also observed evidence of increased anxiety via the Elevated Plus Maze. We found a significant increase in the total amount of mature, adult‐born hippocampal neurons in mice with LRP1KO compared to controls by 9 months. This increased number of neurons was not associated with altered neural stem cell proliferation, but instead was associated with a significant decrease in the apoptosis marker TUNEL in 4‐month‐old mice. Despite a greater number of adult‐born neurons, LRP1KO was associated with reduced dendritic complexity.

**Conclusion:**

Loss of LRP1 in adult neural stem cells increases the number of aberrent adult born neurons in the hippocampus, possibly due to evasion of normal apoptotic mechanisms which occur during neurogenesis. Our results suggest that impairing LRP1 function (such as through interaction with Alzheimer's disease associated proteins) could impair hippocampal neurogenesis to negatively influence hippocampal function. Future studies will examine how LRP1 causes evasion of cell death, and whether Alzheimer's disease associated states impair hippocampal function through LRP1.

